# Generation of a novel three-dimensional scaffold-based model of the bovine endometrium

**DOI:** 10.1007/s11259-023-10130-0

**Published:** 2023-05-08

**Authors:** MC. Díez, S. Przyborski, A. del Cerro, M. Alonso-Guervós, T. Iglesias-Cabo, S. Carrocera, MA. García, M. Fernández, L. Alonso, M. Muñoz

**Affiliations:** 1 Servicio Regional de Investigación y Desarrollo Agroalimentario (SERIDA), Área de Genética y Reproducción Animal. Camino de Rioseco, Deva Gijón, 1225 – 33394 Asturias, Spain; 2 Department of Bioscience, Durham University, Durham, DH1 3LE UK; 3 Optical Microscopy and Image Processing Unit, Scientific-Technical Services, University of Oviedo, Asturias, Spain; 4 Scientific-Technical Services, Statistical Consulting Unit, University of Oviedo, Asturias, Spain; 5 Asociación. Española de Criadores de Ganado Vacuno Selecto Raza Asturiana de los Valles, Asturias, Spain; 6 Matadero Central de Asturias, Asturias, Spain

**Keywords:** Endometrium, Three-dimensional (3D) cell culture, Scaffold, Co-culture

## Abstract

Bovine in vitro endometrial models that resemble tissue function in vivo are needed to study infertility, long-term uterine alterations induced by pathogens and impact of endocrine disruptor chemicals on reproductive function and other reproductive system complications that cause high economic losses in livestock species. The present study aimed to generate an innovative, reproducible, and functional 3D scaffold-based model of the bovine endometrium structurally robust for long term-culture. We developed a multicellular model containing both endometrial epithelial and stromal cells. Epithelial cells organized to form a luminal-like epithelial layer on the surface of the scaffold. Stromal cells produced their own extracellular matrix forming a stable subepithelial compartment that physiologically resembles the normal endometrium. Both cell types released prostaglandin E_2_ and prostaglandin F_2α_ following a treatment with oxytocin and arachidonic acid. Additionally signal pathways mediating oxytocin and arachidonic acid stimulation of prostaglandin synthesis were analyzed by real time PCR (RT-PCR). *Oxytocin receptor* (*OXTR*), *prostaglandin E*_*2*_* receptor 2* (*EP2*), *prostaglandin E*_*2*_* receptor 4 *(*EP4*), *prostaglandin F receptor* (*PTGFR*), *prostaglandin E synthase* (*PTGES*), PGF-synthase (*PGFS*) and prostaglandin-endoperoxide synthase 2 (*COX-2*) expression was detected in both control and treatment groups, however, only significant changes in abundance of *OXTR* mRNA transcripts were found. The results obtained by this study are a step forward in bovine in vitro culture technology. This 3D scaffold-based model provides a platform to study regulatory mechanisms involved in endometrial physiology and can set the basis for a broader tool for designing and testing novel therapeutic strategies for recurrent uterine pathologies.

**Supplementary information:**

The online version contains supplementary material available at 10.1007/s11259-023-10130-0.

## Introduction

Worldwide, uterine infections associated to undiagnosed persistent inflammation cause high economic losses in livestock species due to infertility (Sheldon et al. 2019), and subsequent undesired culling (estimated as 1.7 billion euros/year in Europe for dairy cow productions, and largely unknown in beef production systems). Additionally, important economic losses are also produced in the livestock industry by the exposure to endocrine-disrupting chemicals (EDCs). EDCs, which are found in a great variety of household and industrial products, cause adverse and irreversible effects on animal reproduction, mainly during -utero embryo development and postnatal (Kotwica et al. 2006; Wrobel et al. 2015; Wrobel and Mlynarczuk 2018).

The biggest challenge in studying these disorders and other uterine pathologies is that, so far, there are only few in vitro animal models of the uterus that accurately recapitulate the intricate tissue architecture of the endometrium, and reproducibility is limited (Felgueiras et al. 2020; Almeida et al. 2022). New reliable, reproducible and easy to use in vitro tissue models are needed to balance the need of safe testing in preclinical reproductive research using animals with the simultaneous public concerns and policy efforts to minimize the use of live animals in scientific research.

Over the last decades, great efforts have been dedicated to proposing the use of advanced in vitro tissue models to bridge the gap between the in vivo situation and conventional oversimplified in vitro two-dimension (2D) monolayer models. A common feature in this new generation of in vitro models is their three-dimension (3D) character and the incorporation of multiple cell types, which modifies physical constraints to cells and allows incorporating in the model cell–cell, and cell-extra-cellular matrix (ECM) interactions, all of which are essential to preserve the cell phenotype, behavior, and differentiation status (Knight and Przyborski 2015). Additionally, the preparation of multicellular 3D in vitro models, is key to emulate the structure and function of tissues in vivo since all organs combine multiple cell types in specific arrangements.

Approaches to 3D culture can be broadly categorized into scaffold-free or scaffold-based culture systems, with scaffolds made from either natural or synthetic materials. There is no one particular solution that currently satisfies all requirements and researchers must select the most appropriate method in line with their needs (Knight and Przyborski 2015, Penarossa et al. 2021; Badr-Eldin et al. 2022). Some applications favor the scaffold- free-form growth environments i.e., organoids. Organoids allow for more representative growth of certain tissues — cancerous tissues, in particular — that naturally grow without strict adherence to a cellular scaffold. Conversely the development of physiological relevant 3D in vitro models of epithelial tissues such as the skin or the intestine, technically more challenging due to their multilayered, multicellular structure, has been addressed using scaffold-based approaches (Costello et al. 2021).

With respect to the 3D in vitro tissue culture of reproductive tissues, scaffold-based culture systems have been developed for several human tissues and full organs (for review see Francés-Herrero et al. 2022). The most recent studies have focused on the development of in vitro models to better understand uterine microenvironment, both in physiological and pathological contexts (Almeida et al. 2022). Numerous studies have established the feasibility to use natural or synthetic biomaterials and decellularized tissues as scaffolds for 3D uterine reconstruction in humans, rabbits, and rodents (Almeida et al. 2022). On the contrary, the reduced number of studies conducted in large animals i.e., pigs and sheeps, used on most occasions uterine decellularized scaffolds with the aim to develop useful techniques and models for the regeneration and repair of lesions and for transplantation studies in humans [12–16]. Of interest, the study of Mackintosh et al. (2015) established the only 3D model composed of bovine endometrial epithelial and stromal cells using a tailor-made electrospinned polyglycolide scaffold.

To our knowledge, there is currently no 3D tissue model of the bovine endometrium generated using commercially available off-the-shelf consumables to create a robust and reproducible biomimetic model. Developing such a model faces challenges that include amongst others choosing the most appropriate: i) source of cells i.e., primary cells vs. cell lines, ii) type of scaffold i.e., natural vs. synthetic scaffolds and iii) use of exogenous extracellular matrix components.

The present study aimed to generate a reproducible functional reconstruction of the bovine endometrium structurally robust for long term-culture. Thus, we developed a co-culture model of bovine endometrial epithelial and stromal cells using a commercially available highly porous polystyrene scaffold with appropriate pore sizes to support long term growth and cell to cell contact of epithelial and stromal endometrial cells. Stromal cells produced their own extracellular matrix forming a stable subepithelial compartment that physiologically resembles the normal endometrium. Functionality of the 3D bovine endometrial tissue model was tested by measuring the accumulation of prostaglandin E2 (PGE_2_) and prostaglandin F_2α_ (PGF_2α_) following treatment with oxytocin (OT) and arachidonic acid (AA). Additionally changes in gene expression of *oxytocin receptor* (*OXTR*), *prostaglandin E2 receptor 2* (*EP2*), *prostaglandin E2 receptor 4* (*EP4*), *prostaglandin F receptor* (*PTGFR*), *prostaglandin E Synthase* (*PTGES*), *PGF-synthase* (*PGFS*) and *prostaglandin-endoperoxide synthase 2* (*COX-2*) were analyzed by RT-PCR.

## Material and methods

All reagents were purchased from SIGMA- Aldrich, except otherwise indicated.

### Isolation of endometrial cells

Uteri of the early-luteal phase (days 1–4 of oestrous cycle) were collected from a local abattoir (Matadero Central de Asturias, Noreña-Spain) from nonpregnant cattle (*Bos taurus*) under 30 months of age, immediately following slaughter. The stage of the reproductive cycle was determined by observation of ovarian morphology as previously described (Ireland et al. 1980). The uteri were transported to the laboratory on ice and kept on ice while processing. The endometrial cells were isolated independently from the uteri of fifteen animals and each experimental replicate (n = 5) used 3 animals on separate occasions. The experiments used technical replicates of, at least, two scaffolds for each treatment and for each animal. Isolation of endometrial epithelial (EECs) and stromal (ESCs) cells was performed as previously described (Murillo and Muñoz 2021) with modifications.

For EECs collection, after external cleaning with saline and 70% ethanol, uterine horns ipsilateral to the corpus luteum were excised, ligated from both ends, and filled with a digestion solution containing 0.6% Dispase II (Roche BR, 4,942,078,001) in Hanks Buffered Saline Solution (HBSS; Sigma). After 60 min incubation at 37 °C, Dispase solution was removed. For epithelial cells collection, the horn was longitudinally excised and opened. The inner surface was scratched with a sterile scalpel. EECs were collected in 10% heat-inactivated fetal bovine serum (FBS) in HBSS before centrifugation at 700 g for 7 min. The supernatant was discarded and the cell pellet was re-suspended in culture media (CM) containing 10% FBS, streptomycin (50 μg/ mL), penicillin (50 IU/ mL) and amphotericin B (2.5 μg/ mL) in DMEM-F12 (Sigma).

Subsequently, for ESCs collection, endometrial tissue strips were removed, minced, and digested in HBSS containing BSA (1 mg/mL), collagenase II (0.5 mg/mL), trypsin EDTA (2.5 BAEE units/mL) and DNAse I (0.1 mg/mL). After digestion, the suspension was filtered through a 40 mm mesh cell strainer (Thermofisher Scientific), centrifuged 7 min at 700 g and the pellet was resuspended in CM.

EECs and ESCs were plated separately in culture flask at 1 × 10^5^ cell/mL. To obtain pure stromal cell populations, CM of ESCs was removed 18 h after plating, which allowed selective attachment of stromal cells (Fortier et al. 1988). The culture media was changed every 48 h until cells reach 80% confluence. All cultures were maintained at 37 °C and 5% CO_2_ in air in a humidified incubator.

Purity of isolated EECs and ESCs was identified by morphology and cell-specific staining (95% positive signal) of cytoskeletal proteins in samples cultured *ex-professo* (Murillo and Muñoz 2021). Primary antibodies to cytokeratin or vimentin in combination with goat Alexa Fluor plus 488 anti-rabbit (Invitrogen) and goat Alexa Fluor plus 594 anti-mouse secondary antibodies (Invitrogen) were used to examine epithelial and stromal markers, respectively (see Table 1 for detailed antibody information).

**Table 1:** Antibodies used for immunofluorescence staining

Primary antibody	Clone	Isotope	Working dilution	Supplier	Reactivity
Anti-Vimentin mAb	Monoclonal	IgG1	1:200	SIGMA V6630	Bv, R, Rb, C, P, Fe, Ha, G, Mo, Ch, Ho,
Anti-Cytokeratin mAb	C11	IgG1 & IgG2a	1:400	SIGMA C2931	Bv, Ms, R, H, F, K
Anti E-cadherin pAb	Polyclonal	IgG	1:200	MyBiosource MBS820460	Bv, R, H, Rb, C, NHP, P, Z
Anti-Collagen III pAb	Polyclonal	IgG	1:500	Abcam Ab7778	Bv, Ms, R, H
Goat Alexa Fluor Plus 488 anti-rabbit pAb	Polyclonal	IgG	1:1000	Invitrogen A32731	Rb
Goat Alexa Fluor® 594 anti-mouse pAb	Polyclonal	IgG	1:1000	Invitrogen A-11032	Ms

mAb: monoclonal antibody, pAb: polyclonal antibody, Bv: bovine; Ms: mouse; R: rat; H: human; F: frog; K: kangaroo; Rb: rabbit; C: canine, NHP: non-Human Primate; P: Porcine; Z: Zebrafish/Fish; Fe: feline; Ha; G: gerbil; Mo: monkey; Ch: chicken; Ho: horse

### 3D Co-culture of endometrial stromal and epithelial cells

To develop a viable co-culture model, robust ESCs foundations were first developed onto which EECs were then seeded. Alvetex™ Scaffold inserts (Reprocell Europe) were prepared according to manufacturer’s instructions and use in combination with 12 well plates. For this, 2.0 × 10^6^ ESCs were seeded onto the inserts in 50μL CM and incubated at 37 °C in a humidified atmosphere of 5% CO_2_ for 1 h. Then, culture media supplemented with 100 µg/mL ascorbic acid were subsequently applied to the bottom of each well to gently flood the insert prior to incubation for a further 21 days changing media every 48 h, to allow the formation of a subepithelial compartment.

To establish an endometrial equivalent, 4.0 × 10^5^ EECs were applied to the subepithelial compartment in 50 μL CM and incubate for a further 3 h. Following, CM supplemented with 100 µg/mL ascorbic acid was applied to the outer side of each well to gently flood the insert prior to incubation for a further 14 days changing media every day.

On days 7, 14 and 21, 28, and 35 cell cultures were assessed for histological analysis using hematoxylin and eosin (H & E) staining, or immunofluorescence (IF) analysis.

### Fixation, paraffin embedding and H & E staining

3D in vitro cell cultures (both 3D stromal monocultures and epithelial and stromal co-cultures from now onwards refer as **3D bovine endometrial constructs, 3D BECs)** were washed in PBS prior to fixation in 4% paraformaldehyde for 20 min. Subsequently, 3D BECs were paraffin embedded following the protocols previously described for cell co-culture models on Alvetex™ scaffolds (Costello et al. 2019). Samples were sectioned into 5 µm slices, deparaffinized, rehydrated to distilled water before being stained in Mayer’s Hematoxylin for 5 min. Slides were then washed in tap water and counter-stained in Eosin followed by dehydration to 100% ethanol. Slides were cleared twice in Histoclear and mounted in DPX (Eukitt) before imaging on an Olympus BX53F2 microscope.

Some fixed 3D BECs were stored in PBS at 4 °C until being process for whole mount immunofluorescence (IF) staining.

### Immunoflourescence staining and imaging

For IF examinations, paraffin embedded samples were sectioned at 5 µm and mounted on glass slides. Primary antibodies used for IF included mouse anti-cytokeratin, mouse anti-vimentin, rabbit anti-E cadherin (MyBiosource), rabbit anti-Collagen III (Abcam). Secondary antibodies included goat Alexa Fluor plus 488 anti-rabbit and goat Alexa Fluor plus 594 anti-mouse (see Table 1 for detailed antibody information).

After paraffin removal and rehydration, sections were permeabilized with 0.5% (v/v) Triton X-100 in PBS for 20 min at room temperature (RT), washed with 0.1% Tween.20 in 0.1 M PBS (Washing solution; WS) and blocked for 1 h at RT with 10% goat serum (Abcam) in PBS (Blocking solution; BS). Primary antibodies were diluted in BS and incubated overnight at 4 °C. Secondary antibodies were diluted in BS and incubated for 2 h at RT in darkness. Finally, after further washing in WS for 15 min, sections were incubated for one minute in DAPI reagent (2.3 mg/mL, Sigma-Aldrich, Spain) to stain nuclei and mounted using Vectashield Mounting medium (Vector Labs, USA). Negative controls using only secondary antibodies were performed in parallel.

Whole-mount preparations of the 3D BECs were analyzed by double IF to further characterize the cellular architecture within 3D BECs. The procedure was carried out as described above for tissue sections, with minor modifications (for detailed information see Supplementary Fig. 1).

Confocal microscopy images were captured using a Leica TCS SP8 X confocal microscope (DMI6000 inverted fluorescence microscope; Leica Microsystems). Images were observed with 20x/0.75 and 40x/1.30 oil immersion objectives and data collected using the Leica Application Suite X software platform. A stack of images was taken using sequential acquisition and 1 µm z-steps. Compensation tool was applied to acquired cells placed in deeper positions the 3D BECs. Image processing was performed using Leica LAS X 3D Viewer (Leica Microsystems).

### Treatment of 3D bovine endometrial model

To examine the physiological response of endometrial cells, 3D BECs were treated for 24 h with OT (100 nm) and AA (100 µm), following the protocol previously described by MacKintosh et al. (2015) to induce prostaglandin synthesis. Treatment was applied on day 28 (D28) of culture to both the apical and basolateral compartments separated by the insert containing the 3D BEM. The inner chamber of the well insert was filled with 400 µL CM and the outer well compartment (i.e., underneath the insert) was filled with 1800 µLCM. These volumes are aimed at achieving ‘above and below separately’ feeding regime (see supplementary figure 2). To analyze the effects of treatment on prostaglandin secretion, the 3D BECs were incubated with OT + AA for 24 h and the apical and basolateral supernatants were collected at 18 h and 24 h and analyzed separately.

### Prostaglandin E_2_ and Prostaglandin F_2α_ analysis

Apical and basolateral supernatants were analyzed for PGE_2_ and PGF_2α_ concentration using an ELISA method following supplier recommendations (Prostaglandin F_2α_ EIA Kit, 516,011; Prostaglandin E_2_ EIA Kit, 514,010; Cayman). The supernatants were diluted in the provided buffer as appropriate. Briefly, 50 µL of diluted sample were added to each experimental well, followed by 50 µL of PGF_2α_ or PGE_2_ Ache tracer and 50 µL of PGF_2α_ or PGE_2_ EIA antiserum. Subsequently, ELISA plates were incubated for 18 h at 4 °C. Plates were washed five times with the provided wash buffer, and 200 µL of Ellman’s reagent were added to each well. Finally, the plates were covered with a plastic film and allowed to develop in the dark. The absorbance was measured at 450 nm in a microplate reader. Positive and negative sera controls were added to each microtiter plate for normalization. Controls and sera samples were analyzed in duplicate. Each tissue culture supernatant sample was assayed in duplicate. The mean of the duplicate values was used for statistical analysis. The concentration of PGE_2_ and PGF_2α_ in each sample was interpolated from the standard curve. The intra- and inter- assay coefficient of variations for prostaglandins (PGs) analysis were: PGF_2α_—9.1% and 9.2% and PGE_2_ -3.7% and 9.7% respectively.

### Statistical analysis

Linear Mixed Models were built to study the differences between the OT + AA treatment applied in the different endometrial cell populations at 18 h and 24 h, which included the fixed effects of treatment, treatment duration and cell compartment, while uteri and treatment duration were considered as random effects. The level of significance was set at 0.05.

Analysis was performed using R version 4.1.3.(R Development Core Team Ref 2022), and the lmerTest library (Kuznetsova et al. 2017).

### RNA extraction, reverse transcription, and RT qPCR

Total RNA was extracted from cells cultured in Alvetex™ Scaffolds using the RNeasy mini kit (Qiagen, Germany) at 24 h post-treatment, following the protocol described by Reprocell (https://www.reprocell.com/resources/protocols#alvetex-protocols-1). All samples were DNase treated using the Ambion DNA-free kit (Ambion, USA) and RNA quantity and integrity was checked using a Qubit fluorometer with the Qubit RNA XR and RNA IQ assay kits respectively (Invitrogen, USA). cDNA was then prepared using a NZY First-Strand cDNA Synthesis kit (NzyTech, Portugal). RT-PCR was performed using the Step-One Plus Real Time PCR System and Power UP SYBR Green PCR Master Mix (Applied Biosystems, USA). The reaction mixture for amplification consisted of 2 µl of diluted cDNA (1:20), 10 µl of SYBR Green PCR Master Mix and 300 nM of each primer in a final reaction of 20 µl. The primers employed in these assays are listed in Table 2. RT qPCR cycling conditions consisted of two initial stages at 50 °C for 2 min and 95 °C for 2 min, followed by 40 amplification cycles (95 °C for 3 s and 60 °C for 30 s). To confirm product specificity, melting curve analyses were performed immediately after each PCR amplification. Non-template controls were run for each gene and all the qPCR reactions were performed in triplicate. All Ct values from the qPCR were transformed into normalised relative quantities (NRQ) using qBase plus software version 3.3 (Biogazelle), which is based on a generalized model of the 2^−ΔΔCt^ approach with correction of amplification efficiency (Hellemans et al. 2007). For normalization SUZ12 and C2ORF29 were chosen as reference genes according to Walker et al. (2009). For graphical representation, results from qBase (calibrated NRQ values) were processed in R software. The results were statistically analysed using a Mann–Whitney U test to compare experimental and control groups with significance reported for p < 0.05.

**Table 2:** Primers used for the real-time qPCR analysis of cells cultured in Alvetex™ Scaffolds. Suppressor of zeste 12 protein (SUZ12), CCR4-NOT transcription complex subunit 11 (C2ORF29), oxytocin receptor (OXTR), prostaglandin E synthase (PTGES), prostaglandin F synthase (PGFS), prostaglandin E2 receptor subtype 2 (EP2), prostaglandin E2 receptor subtype 4 (EP4), prostaglandin F_2_ receptor (PTGFR), Prostaglandin-endoperoxide synthase 2 (COX-2)

Gene symbol	Product size (bp)	Accession No.	Reference
*Endogenous control genes*			
* SUZ12*	130	XM_582605	Walker et al. 2009
* C2ORF29*	64	XM_582695	Walker et al. 2009
*Genes of interest*			
* OXTR*	96	NM_174134	Walker et al. 2009
* PTGES*	201	NM_174443	Herath et al. 2009
* PGFS*	250	NM_001035367	Herath et al. 2009
* EP2*	246	AF539402	Herath et al. 2009
* EP4*	226	AF539403	Herath et al. 2009
* PTGFR*	151	NM_181025	Zhang et al. 2018
* COX-2*	124	NM_174445	Fu et al. 2020

## Results

**Fig. 1:**
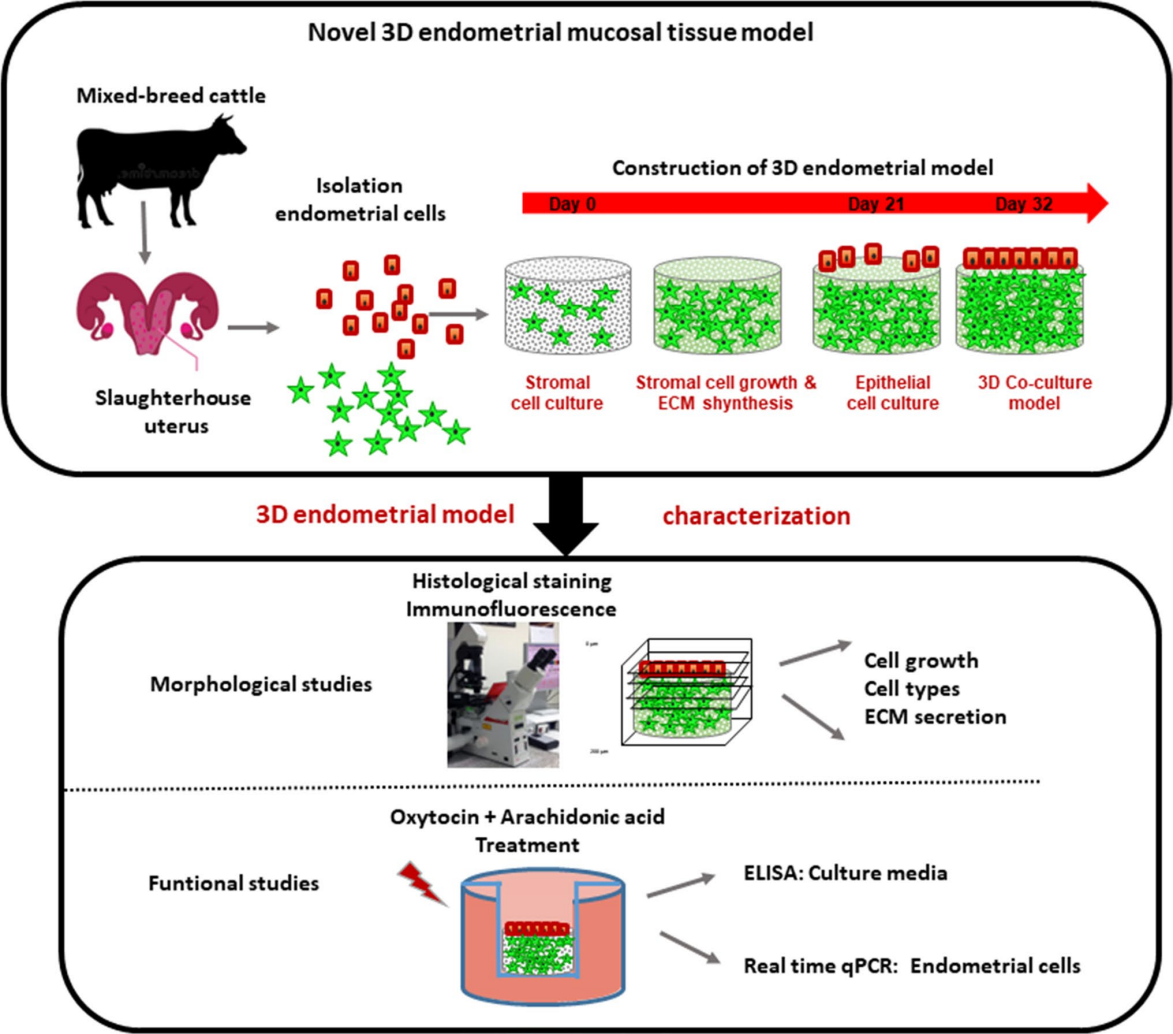
Diagrammatic representation of the construction of the 3D endometrial model and functional assays performed

A general scheme of the development of the 3D scaffold-based bovine endometrial construct, including the preparation of a robust subepithelial/stromal compartment and seeding of epithelial cells, the time course the endometrial cell co-cultures and their morphological and functional characterization is shown in Fig. 1.

### Generation of a 3D scaffold based bovine endometrial model: co-culture of primary endometrial stromal fibroblasts and epithelial cells

**Fig. 2:**
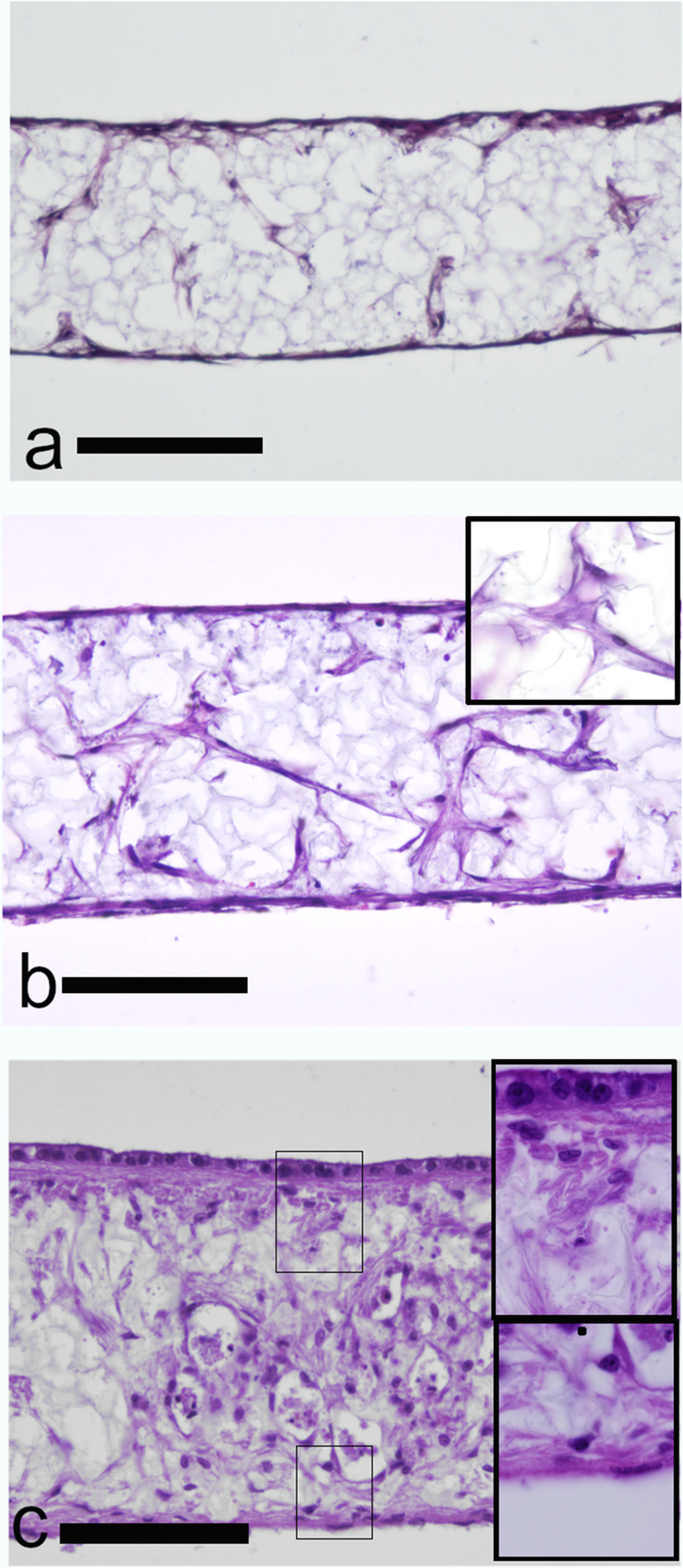
Validation of subepithelial/stromal and epidermal structure in full-thickness bovine endometrial tissue equivalents. (**a**, **b**) Representative photomicrographs of haematoxylin and Eosin (H&E) stained Alvetex™ seeded with bovine endometrial fibroblast for: (**a**) seven days (**b**) fourteen days. (**c**) Seven days after epithelial cell seeding, a single cuboidal monolayer of epithelial cells was established

Commercially available highly porous polystyrene scaffolds were used to create a bovine 3D BEC in the absence of any exogenous animal matrix component. Pretreatment of scaffolds was performed according to manufacturer instructions by immersion in 70% ethanol. Subsequently, primary bovine endometrial stromal fibroblasts isolated following established protocols in our laboratory (Murillo and Muñoz 2021) were seeded onto the upper surface of the scaffolds and cultured for three weeks at 37 °C in a humidified atmosphere of 5% CO_2_. Histological analysis of scaffold cross sections on day seven (D7), fourteen (D14) and twenty-one (D21) demonstrated that by D7 ESCs were predominantly attached on the scaffold edges with little evidence of cellular infiltration into the scaffold (Fig. 2a). However, during the second and third week of culture, infiltrated fibroblasts within the scaffold started to grow and showed a large, flat, elongated (spindle-shaped) morphology (Fig. 2b).

**Fig. 3:**
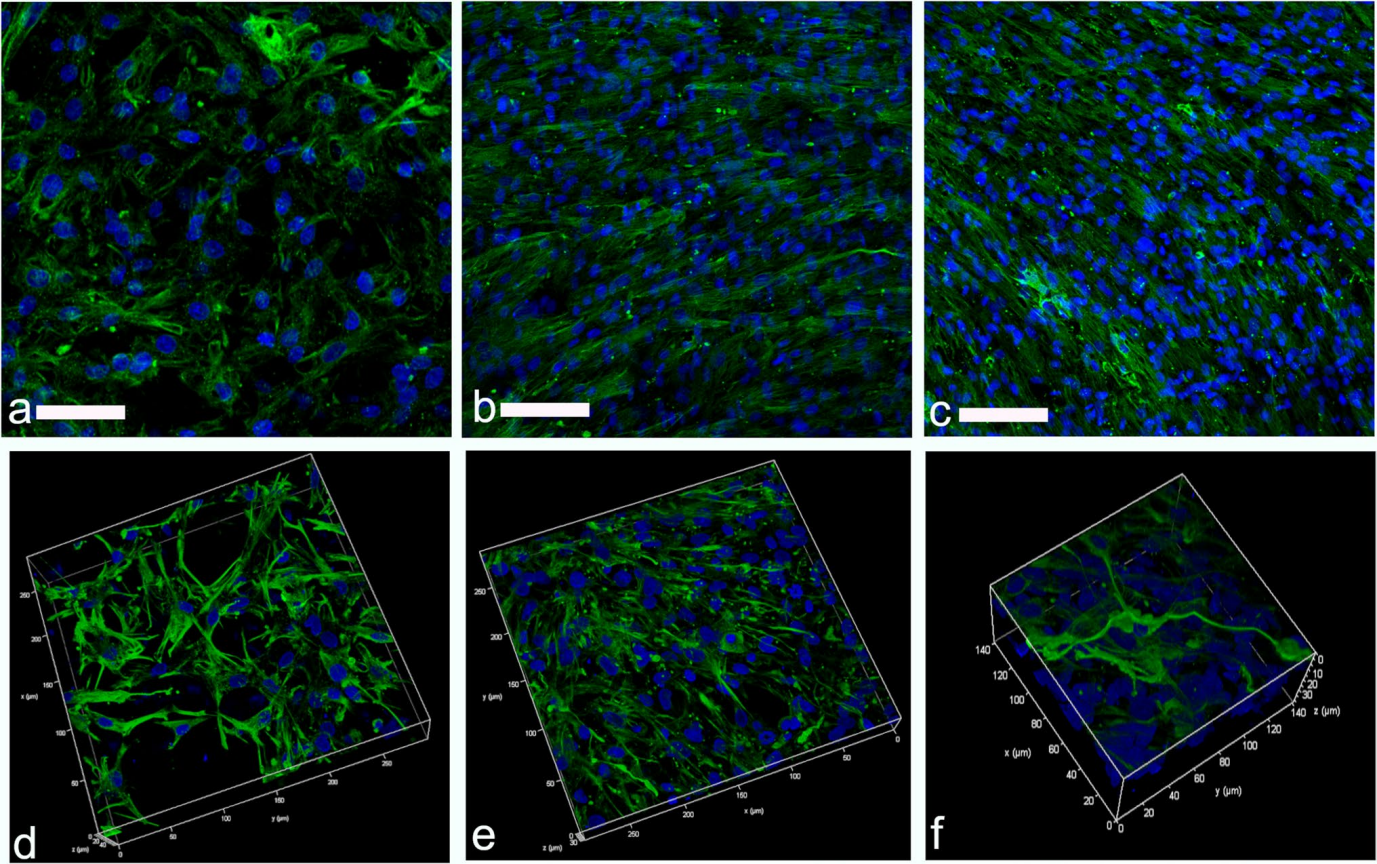
Expression of vimentin fibroblast marker in subepithelial stromal cells over time. Representative fluorescent confocal photomicrographs for the expression of vimentin (green) in single confocal laser microscopy section at: (**a**) seven days, (**b**) 14 days and (**c**) 21 days after fibroblast seeding on Alvetex™; and high-quality 3D images obtained by processing the optical section stacks with volume render and surface display parameters in microVoxel at: (**d**) seven days, (**e**) 14 days and (**f**) 21 days after fibroblast seeding. Nuclei of the cells were stained using DAPI (blue)

Confocal microscopy in combination with whole-mount immunofluorescence staining of ESCs seeded scaffolds, verified the expression fibroblast lineage specific marker vimentin (Fig. 3a-f) and revealed that fibroblasts had created by day 21 a robust foundation for epithelial cells to grow (Fig. 3c). Fibroblasts grown within the scaffold frequently showed long cytoplasmic protrusions over 100 µm (Fig. 3f).

**Fig. 4:**
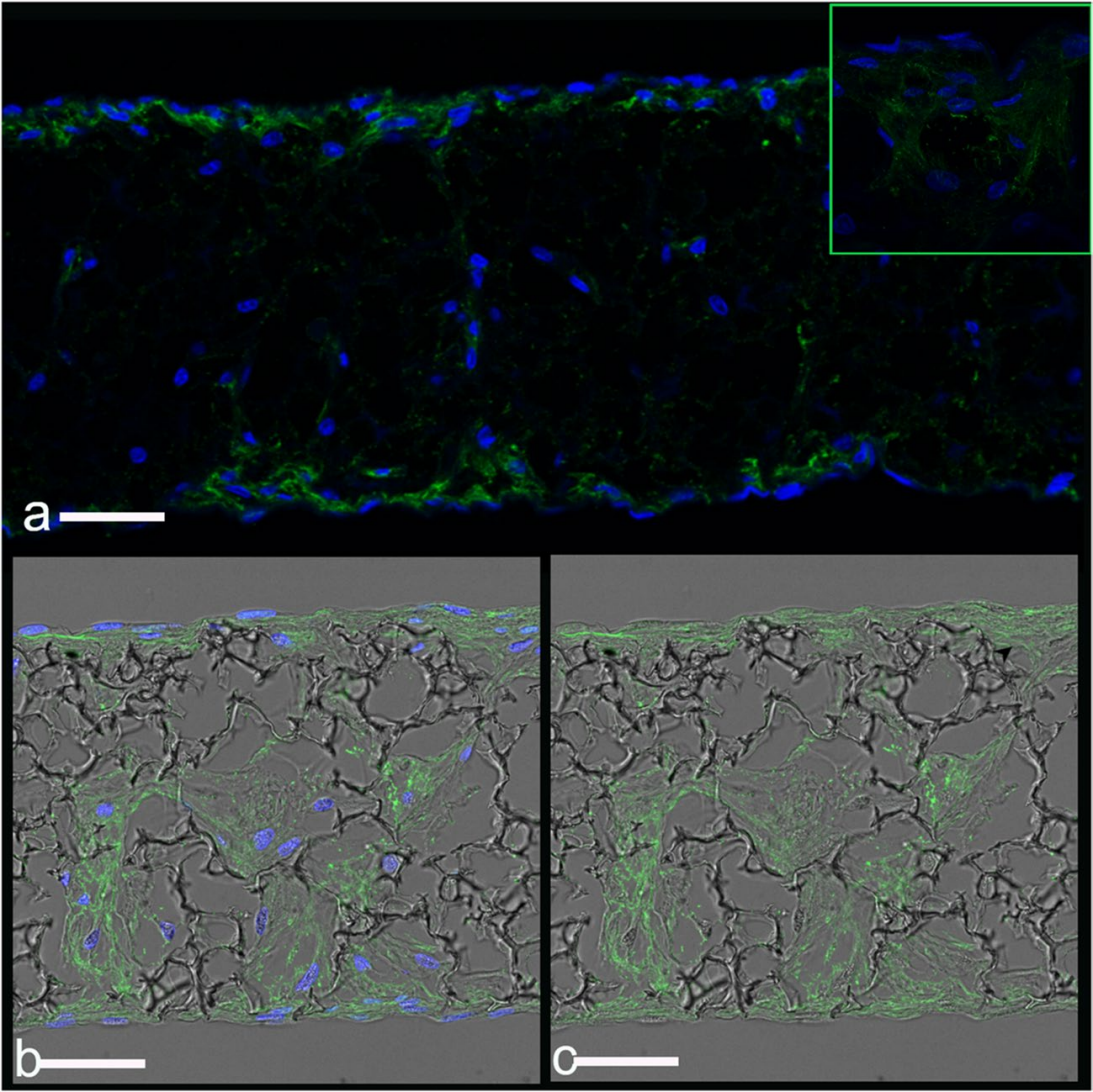
Extracellular matrix component type III collagen in subepithelial stromal compartment. (**a**) Representative fluorescence photomicrograph of type III collagen (green) expression of cross-sectional section of stromal seeded scaffolds on day 21 and (**b** and **c**) combination of fluorescence image and transmission image with confocal microscopy was used to visualized Collagen III expression (green) on Alvetex™ scaffold. Images (**a**) and (**b**) with nuclei of the cells stained with DAPI (in blue). (**c**) image (**c**) without DAPI to improve visualization of collagen III deposition over scaffold

Immunofluorescence staining also revealed the expression of Collagen III, a major structural protein of the extracellular matrix, critical for the long-term maintenance endometrial equivalent (Fig. 4a-c). Collagen III, coated small pores and connected material interfaces (Fig. 4c), which provided a larger surface for cell migration and cell colonization. The presence of both fibroblasts and ECM components is representative of a sub-epithelial tissue-like layer and results in the overall structure of the in vitro co-culture being like the in vivo endometrium.

**Fig. 5:**
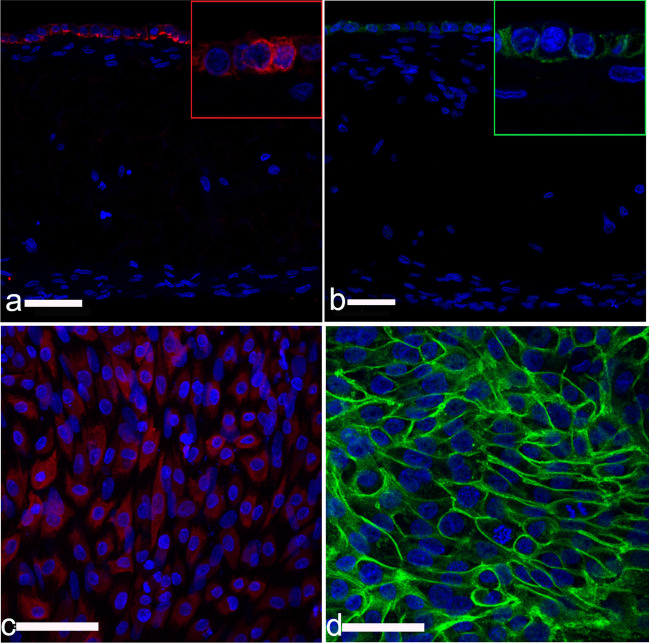
Immunohistochemistry images of co-cultured stromal and epithelial cells seeded on Alvetex scaffolds on day 32 of culture. Cytokeratin (**a**, red) or E-cadherin (**b** green) expression by epithelial cells of cross sectional sections of the scaffold. Both epithelial markers are restricted to the monolayer of epithelial cells found on top of the stromal compartment. Representative fluorescent confocal photomicrographs for the expression of cytokeratin (**c**) and E-cadherin (**d**) were obtained at the top cellular monolayer of the endometrial construct. For all images, DAPI was used as nuclear stain (blue)

Next, we evaluated the ability of EECs to create an epithelial cell monolayer on top of the fibroblast populated scaffolds. EECs (own protocols, Murillo and Muñoz 2021) were seeded on top of the stromal cells and cultured for a further week. Epithelial cells with a cuboidal morphology built a closed monolayer overlaying the stromal cell mass after seven days of co-culture (Fig. 2c). Additional insight into the architectural arrangement of the epithelial monolayer was revealed by the expression of epithelial markers cytokeratin and E-cadherin. Bovine endometrial epithelial cells grown for seven days, showed cytokeratin expression localized throughout the epithelial cell cytoplasm (Fig. 5a, 5c), while E-cadherin appeared to be localized to the lateral membranes (Fig. 5b, 5d).

**Fig. 6:**
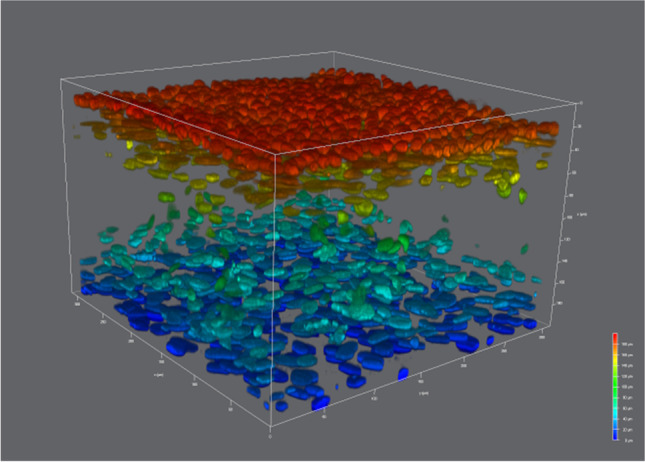
Depth-color coded projection of co-cultured stromal and epithelial cell nuclei seeded on Alvetex™ scaffolds on day 35 of culture. 3D reconstruction of confocal microscope images of epithelial and stromal cells co-cultivated on Alvetex™ scaffold for 35 days. Compensation tool during confocal images acquisition enabled to visualize cell nuclei placed in deeper positions within the scaffold. Cell nuclei have been color-coded based upon depth (DAPI stain)

After 35 days of culture (twenty-one day of stromal culture + 14 days epithelial/stromal co-culture), examination of nuclear morphology by fluorescent staining and Immunofluorescence analysis coupled to confocal microscopy and 3D volume reconstruction software confirmed the multi-layer, multi cellular organization of the 3D BECs. Using the depth-color code tool of the Leica confocal microscope software, a tight monolayer of cell nuclei colored in red exhibiting solid spherical morphology was visualized at the surface of the scaffold. Additionally, below the surface, elongated-ovoid cell nuclei colored in yellow, green, and blue were distributed all through the sub-epithelial compartment (Fig. 6).

**Fig. 7:**
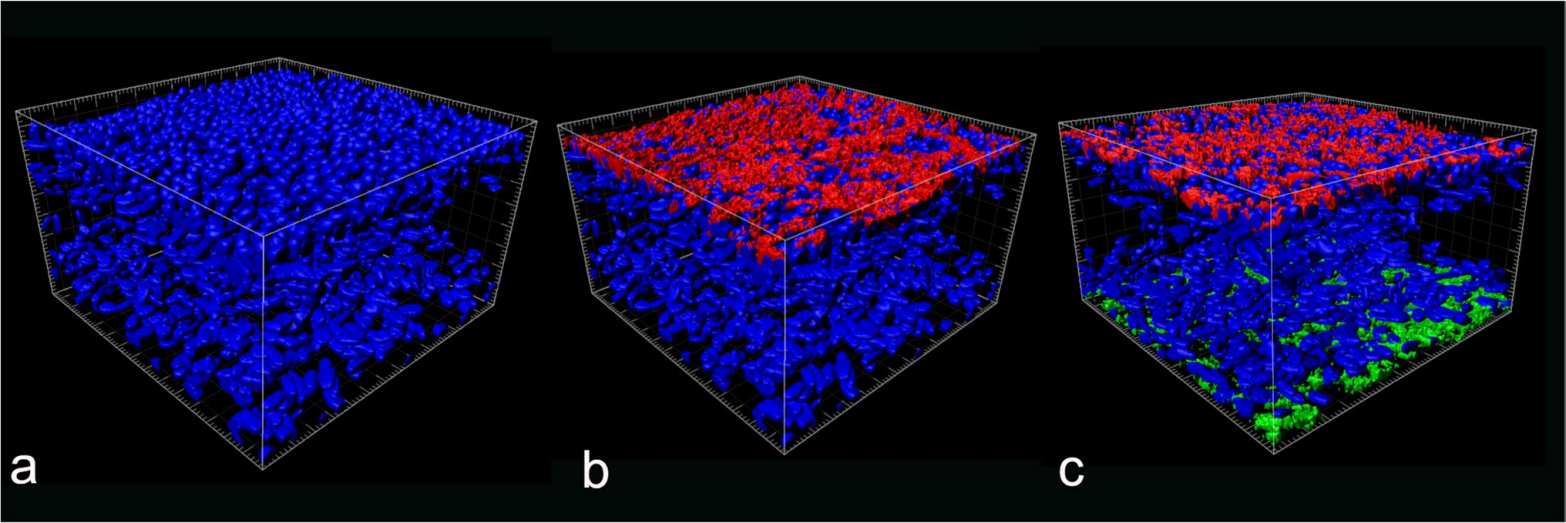
3D rendering illustrating the distribution of epithelial and stromal cells within the co-culture on day 35 of culture. **a**) 3D BECs cell nuclei with DAPI stain (blue). (**b** and **c**) Cytokeratin (red stain) is localized at the epithelial cell layer in the upper surface of the 3D endometrial co-culture model. (**c**) Vimentin (green stain) is expressed by fibroblast located at the bottom layers

The expression of cytokeratin, was restricted to cells located in the surface layer (Fig. 7b, 7c), whereas vimentin immunoreactivity was found in cells located in bottom compartment (Fig. 7c). All the features described above recapitulated the morphological characteristics of the endometrial in vivo, suggesting that the 3D BECs allows a high degree of cell differentiation toward an endometrium cell phenotype.

### Functionality of the 3D endometrial construct

#### Endometrial cells produce prostaglandins in response to oxytocin and arachidonic acid treatment

**Fig. 8:**
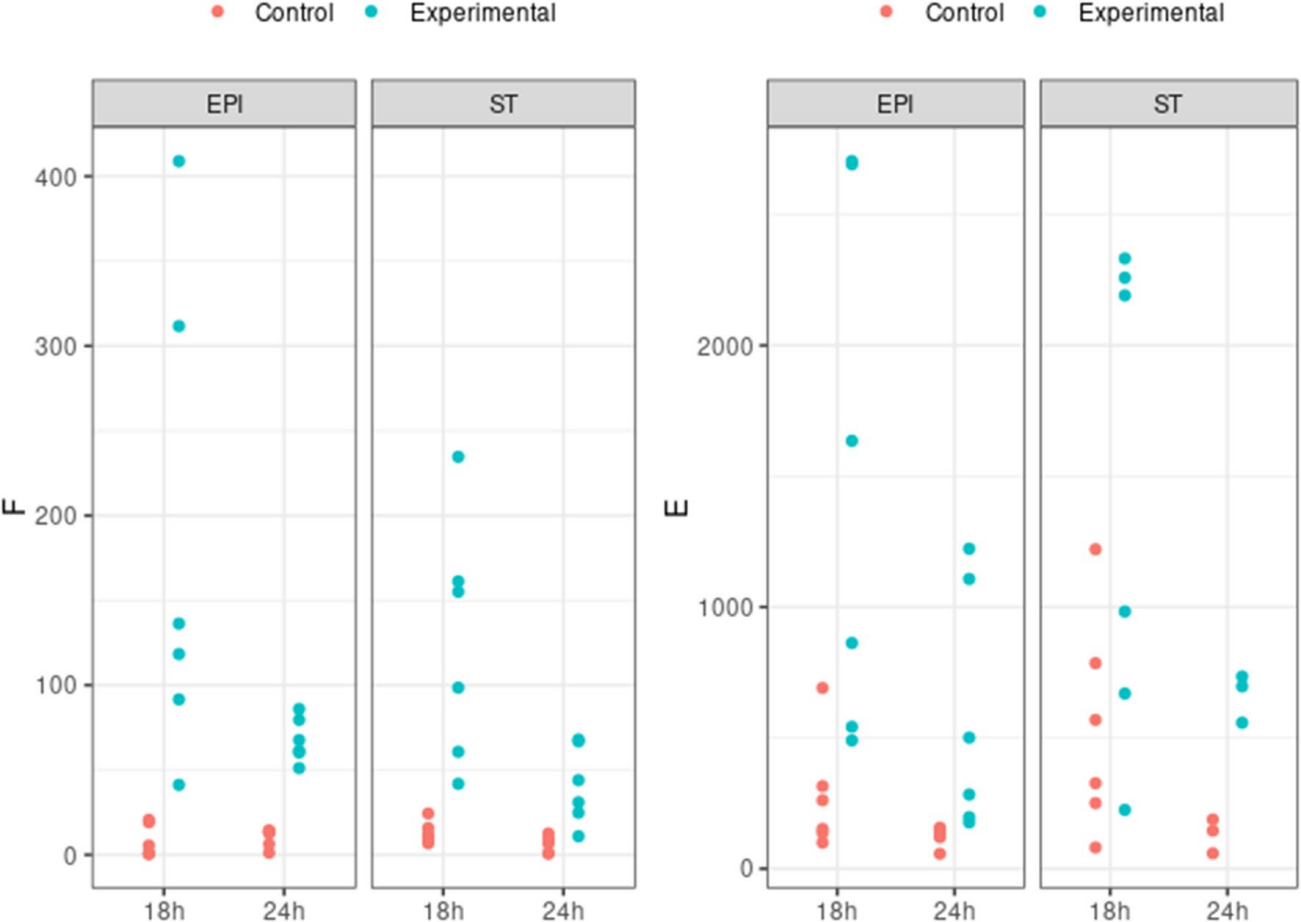
Prostaglandin accumulation of epithelial and stromal cell co-cultured on Alvetex™ scaffold and treated with oxytocin plus arachidonic acid. Accumulation of (**a**) PGF and (**b**) PGE following 18 h or 24 h treatment. Supernatants from the apical (EPI) and basal compartments (ST) were analyzed separately. Prostaglandin accumulation differed significantly between treated and control (P < 0.05)

To investigate the effect of OT and AA on the 3D BEC, the concentration of PGF_2α_ and PGE_2_ was measured at 18 and 24 h post treatment in treated and non-treated control samples (Fig. 8, Supplementary Table 3). Additionally, to study which endometrial cells were associated with the OT and AA induced changes in PGs concentrations, the cultured media recovered from the apical and the basolateral compartments were analyzed separately.

A total post treatment increase in the concentration of PGE_2_ and PGF_2α_ (taking together 18 + 24 h results) was observed (PGE_2_: p < 0.001, β = 95.2; PGF_2α_: P < 0.001, β = 811,23). The effect of treatment changes with time, observing a significant decrease at 24 h with respect to 18 h (PGF_2α_: p = 0.004, β = -51.2; PGE_2_: p = 0.001, β = -615.51). Unexpectedly, no global significant differences were observed between the apical and basolateral compartments (PGF_2α_, p = 0.215; PGE_2_ p = 0.898).

#### Relative abundance of *EP2*, *EP4*, *PTGFR*, *OXTR*, *PGES,PGFS *and *COX2*

**Fig. 9:**
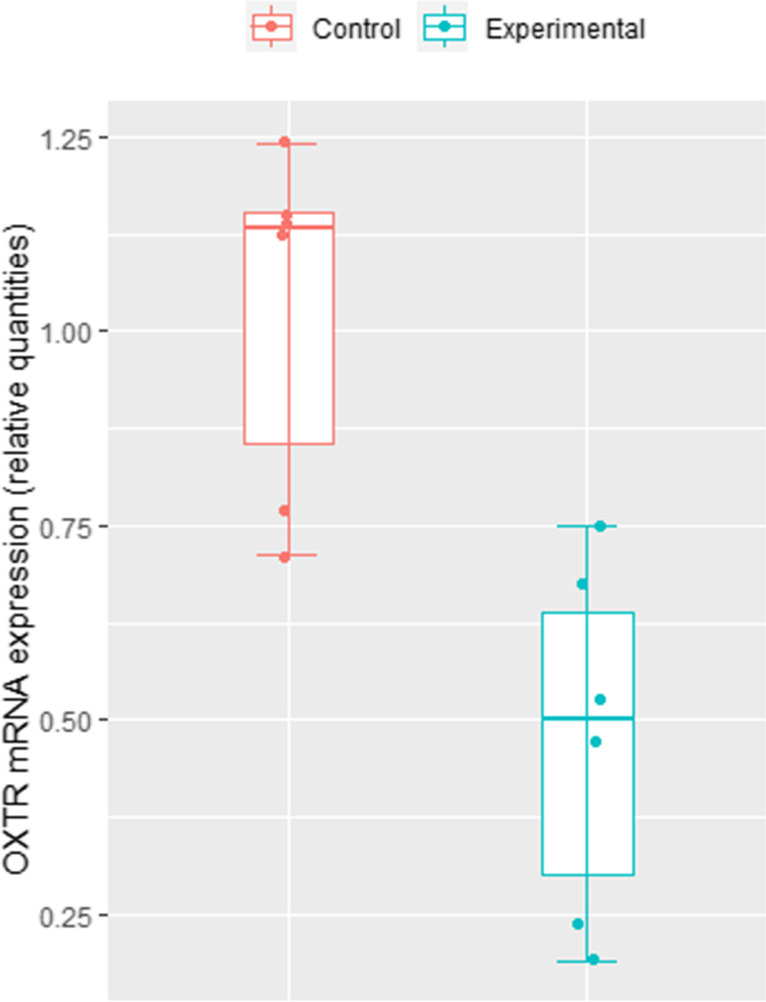
*Oxytocin receptor *mRNA expression by epithelial and stromal cell co-cultured on Alvetex™ scaffold and treated with oxytocin plus arachidonic acid at 24 h. Relative mRNA expression results for *OXTR* in control and experimental groups when normalized with endogenous control genes *SUZ12* and *C2ORF29*. *OXTR* expression was significantly decrease in the experimental group *vs*. the control group (p < 0.05)

To determine the effects of OT and AA on PG synthesis, there was evaluation of prostaglandin E_2_, prostaglandin F_2α_ and oxytocin receptors (*EP2, EP4*, *PTGFR, OXTR*), prostaglandin E_2_ and F_2α_ synthases (*PTGES* and *PGFS)* and *COX2* mRNA transcripts in epithelial and stromal cells recovered from the 3D BECs after 24 h treatment by real-time qPCR. mRNA transcripts of all the genes tested were detected in the cells, but there was no effect of OT + AA treatment on abundance of *EP2, EP4*, *PTGFR, PTGES, PGFS* and *COX2* mRNA (Supplementary Fig. S3). However, the relative abundance of *OXTR* mRNA significantly decrease in treated 3D BECs samples in comparison to control (Fig. 9*p* < 0.05).

## Discussion

The endometrium, the innermost layer of the uterus, is responsible for uterine receptivity, early embryo development, hormonal production maintenance of pregnancy and serves as protection barrier against external agents. Considering the importance of understanding human and animal endometrial physiopathology, and given ethical and practical limitations of obtaining and studying the endometrium, a great effort is being undertaken to develop 3D in vitro models that better mimic its complex, multicellular architecture and function.

At the present time, most 3D cell culture models of the endometrium have been developed for humans or laboratory animals using scaffolds produced from natural biomaterial such as Matrigel, collagen and other natural polymers (Almeida et al. 2022). This strategy enables to recreate the intimate communication between the tightly-bonded epithelial cells and the matrix rich stroma containing fibroblasts that is necessary to develop a functionally normal epithelium (Fitzgerald et al. 2021; Almeida et al. 2022). However, a main limitation of the aforementioned models is that reproducibility is impaired due to the use of complex protocols, inherent batch to batch variations in biomaterial of natural origin, and the difficulties in the long-term co-culturing of epithelial and stromal cells that hinder the use of these models to simulate the estrous cycle or long disease process (Fitzgerald et al. 2021).

Development of 3D endometrial models for livestock animals is scarce despite their potential to study uterine diseases that impair fertility and negatively affect health, welfare and, in the case of production animals, the herd’s economic issues.

In bovine, to the best of our knowledge, only one study has attempted to recreate the histoarchitecture and relationships between different cell types of the endometrium as well as the native ECM (Mackintosh et al. 2015). The reported 3D model involved the design and preparation of a polyglycolide electrospun scaffold that enable to generate a functional reconstruction of the bovine endometrium, but became extremely fragile by days 12–14 of culture making long term culturing unfeasible.

In this study, we therefore aimed to develop an in vitro 3D bovine endometrial tissue model bearing morphological and structural similarity to that of in vivo endometrium. For three weeks primary bovine fibroblasts cells built up and acquired spindle-shape bodies inside the scaffold and on both sides of the 200 µm membrane, indicating that the porosity and stiffness of the scaffolds was suited for cell migration. Fibroblasts produced their own ECM components conforming a stable stromal compartment, key to support the growth of epithelial cells and enable them to form an epithelial monolayer on the surface of the scaffold. Furthermore, the epithelial phenotype of the cells grown on top of the stromal compartment was demonstrated by immunohistochemical detection of E-cadherin, an essential transmembrane protein within adherent junctions that plays pivotal roles in important morphogenetic and differentiation processes during development, and in maintaining integrity and homeostasis in epithelial tissues (Tunggal et al. 2005).

The structure of the 3D bovine endometrial tissue was monitored in-depth over time remaining intact for at least 35 days of culture, which agrees with previous reports describing the use of porous polystyrene scaffolds to bioengineer diverse epithelial tissues that have been grown for several months (Roger et al. 2019; Costello et al. 2021). These results suggest that the used of scaffolds is key to provide, in the long term, a suitable environment in which epithelial and stromal cells growth, differentiate, and function to form close relationships with adjacent cells, thus, creating the equivalent of a thin multilayered tissue in vitro. Overcoming the short lifetime described in other 3D endometrial models (Wang et al. 2012; Mackintosh et al. 2015) is key to study long-term physiological or pathological processes such as host-pathogens interactions during uterine infections or early embryo-maternal communication.

In the present work, the functionality of the 3D BECs was tested by exposing the endometrial constructs to OT + AA for 24 h. Increased accumulation of both PGF_2α_ and PGE_2_ was found in all samples tested, showing a high variability among different endometrial constructs. Such variability may be caused by individual animal differences, or be a result of variable differentiation status of the cells. Accumulation of PGE_2_ in the cultured media recovered from the apical compartment, conditioned by epithelial cells, was greater than PGF_2α_ accumulation. This finding agrees with a previous report that demonstrate that PGE_2_ increase by polarized epithelial cells in a transwell co-cultured with stromal cells was greater than PGF_2α_ increase (MacKintosh et al. 2013). Interestingly, in previous reports, OT + AA treatment stimulated accumulation of more PGF_2α_ than PGE_2_ in monocultures of bovine epithelial cells (Asselin and Fortier 1996; Herath et al. 2006, 2009). These discrepancies sustain the relevance of developing appropriate 3D endometrial models to recreate the multicellular multilayer tissular microenvironment to faithfully study the mechanisms of endometrial function.

Results from the present study indicate that *OTXR* transcripts are downregulated in response to OT + AA treatment. The oxytocin receptor belongs to the G protein coupled receptor family. Studies on a variety of G protein-coupled receptors indicate that their repeated or prolonged stimulation results in the loss of hormonal responsiveness, which is termed desensitization. Several mechanisms such as receptor internalization, post receptor signaling pathway regulation or mRNA downregulation, might be involved in G protein coupled receptor desensitization. In human cultured myometrial cells, desensitization of OT receptors is accompanied by *OXTR* mRNA downregulation after 6 h of exposure to OT (Phaneuf et al. 1997). Moreover, the decrease in OXT receptor binding and mRNA in the myometrium of women receiving oxytocin infusion during labor indicates that homologous receptor desensitization also occurs in vivo. Further investigation is needed to understand if prolonged exposure of bovine endometrial OXTR to OT might lead their desensitization as it has been described in the human myometrium and the clinical importance of this finding.

After OT + AA treatment, however, no significant changes were detected on relative abundance of *EP2, EP4, PTGFR, PTGES, PGFS and COX2 mRNA.* In the present study epithelial and stromal cells were recovered from the 3D BECs using a combination of trypsin and mild agitation that hinder their separation. It cannot be ruled out that the effect of OT + AA treatment on the studied PG signaling related genes has gone unnoticed if it did induce opposite responses on epithelial and stromal cells. It is important to note that in a previous transcriptomal profiling study, using RNA-sequencing in epithelial (luminal and glandular) and stromal cells isolated by LCM in bovine endometrium, Chankeaw et al. (2021) found that a high number of genes were expressed in common in the different bovine endometrial cell types, while lower proportions (5, 7 and 15% for luminal epithelial cells, glandular epithelial cells and stromal cells, respectively) were restricted to each cell type indicating that they code for proteins supporting the functional specialized signature of each cell type. These results demonstrated that separating cell types is more appropriate and possibly less biased to decipher the impacts of any factor than approaches based on full tissue.

To our knowledge, it is unknown if the laser capture microdissection process can be used to retrieve cells embedded in polystyrene scaffolds efficiently, but further investigations analyzing the different types of cells grown on the developed 3D BECs model will help enhance understanding their function.

## Conclusions

In summary, we constructed a novel 3D in vitro bovine endometrial tissue model that enables co-culturing primary epithelial and stromal cells on a 3D matrix for five weeks in a reproducible and consistent manner. We have demonstrated its similarity to native bovine endometrium using detailed morphological and structural analyses. In addition, the 3D endometrial tissue model relies on the formation of endogenous ECM proteins, thus avoiding the use of exogenous components and improving consistency. Importantly, both epithelial and stromal cells are functionally responsive to hormones in the scaffold.

This system is suitable for the introduction of higher levels of complexity including addition of other endometrial cell types such as immune cells to simulate amongst others uterine infections, a very common condition, affecting up to 40% of post-calving cows.

We believe that this model provides a platform for elaborate studies of the regulatory mechanisms involved in endometrial physiology and can set the basis for a broader tool for designing and testing novel therapeutic strategies for recurrent uterine pathologies.

## Supplementary Information

Below is the link to the electronic supplementary material.

Supplementary file1 (DOCX 55 KB)

Supplementary file2 (DOCX 391 KB)

Supplementary file3 (DOCX 22 KB)
