# Supplementary figures and images for: Generation of a novel three-dimensional scaffold-based model of the bovine endometrium
Source: Vet Res Commun. 2023 May 8;47(3):1721–33. doi: 10.1007/s11259-023-10130-0 (PMC10484811; doi:10.1007/s11259-023-10130-0)

**Supplementary Fig. 1**

**
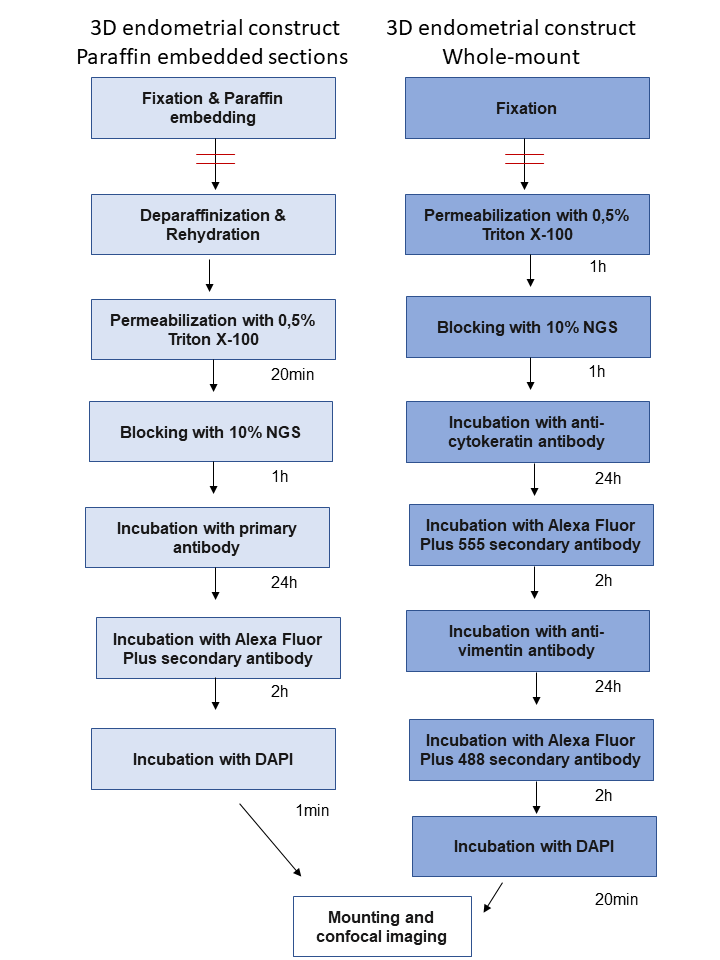
**

Supplement: Supplementary file 1 — Supplementary file1 (DOCX 55 KB) [file 11259_2023_10130_MOESM1_ESM.docx]
