# Supplementary material for: Generation of a novel three-dimensional scaffold-based model of the bovine endometrium
Source: Vet Res Commun. 2023 May 8;47(3):1721–33. doi: 10.1007/s11259-023-10130-0 (PMC10484811; doi:10.1007/s11259-023-10130-0)
Supplement: Supplementary file 2 — Supplementary file2 (DOCX 391 KB) [file 11259_2023_10130_MOESM2_ESM.docx]

**Supplemntary Fig. 2**

Simulation of Alvetex ™ scaffold feeding option used for oxytocin and arachidonic treatment

**
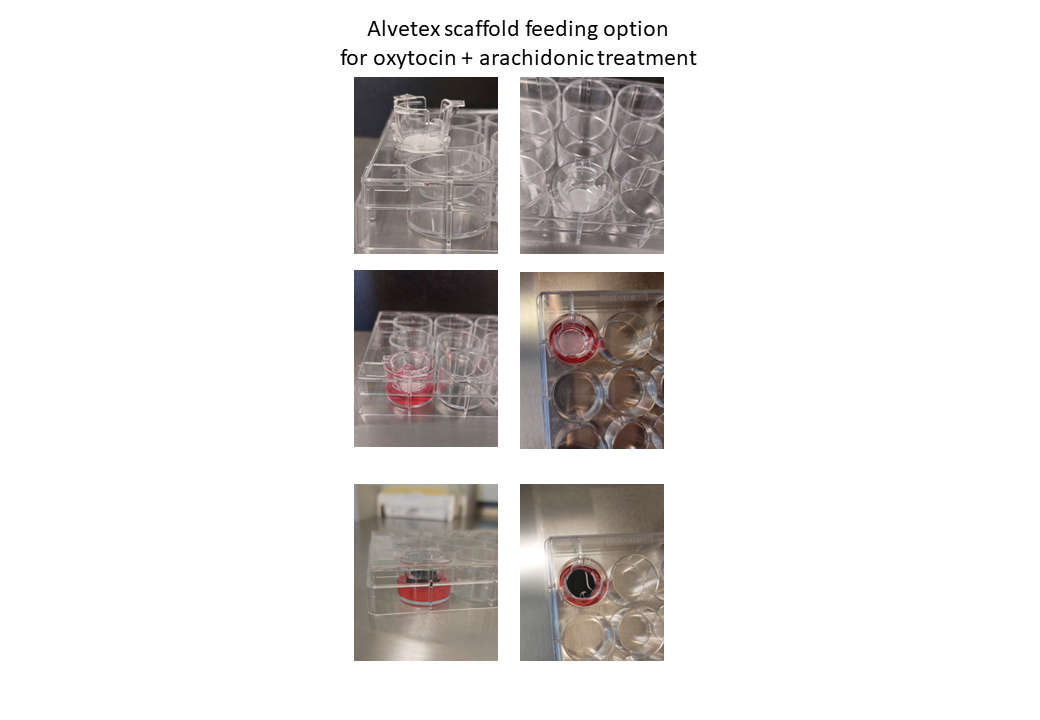
**

The bottom of the insert is just in contact with the media below (red liquid in simulation). The surface of the insert is cover with the media within the insert (blue liquid in the simulation)
