# Supplementary material for: Generation of a novel three-dimensional scaffold-based model of the bovine endometrium
Source: Vet Res Commun. 2023 May 8;47(3):1721–33. doi: 10.1007/s11259-023-10130-0 (PMC10484811; doi:10.1007/s11259-023-10130-0)
Supplement: Supplementary file 3 — Supplementary file3 (DOCX 22 KB) [file 11259_2023_10130_MOESM3_ESM.docx]

**Supplementary Fig. 3**

*Prostaglandin-endoperoxide synthase 2* (*COX-2*), *prostaglandin E2 receptor 2* (EP2), *prostaglandin E2 receptor 4* (*EP4*), *PGF-synthase* (*PGFS*), *prostaglandin E synthase* (*PTGES*), and *prostaglandin F receptor* (*PTGFR*), mRNA expression by epithelial and stromal cell co-cultured on Alvetex™ scaffold and treated with oxytocin plus arachidonic acid at 24h


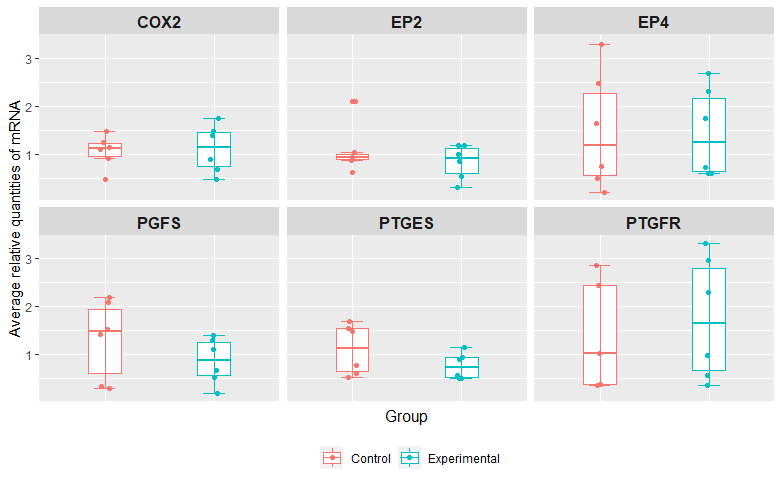


Relative mRNA expression results for *COX2, EP2, EP4,PGFS, PTGES AND PTGFR* in control and experimental groups when normalized with endogenous control genes *SUZ12* and *C2ORF29*. No significant differences were found between the experimental and control groups (p>0.05)
